# Pd-catalyzed decarboxylative Heck vinylation of 2-nitrobenzoates in the presence of CuF_2_

**DOI:** 10.3762/bjoc.6.43

**Published:** 2010-05-03

**Authors:** Lukas J Gooßen, Bettina Zimmermann, Thomas Knauber

**Affiliations:** 1 Department of Chemistry, Organic Chemistry, Technische Universität Kaiserslautern, Erwin-Schrödinger-Strasse, Geb. 54, D-67663 Kaiserslautern, Germany

**Keywords:** carboxylic acids, copper, decarboxylation, Heck vinylation, palladium

## Abstract

A new protocol for the decarboxylative Heck vinylation of benzoic acids is disclosed. In the presence of a catalyst system generated in situ from Pd(OAc)_2_ (2 mol %), CuF_2_ (2 equiv), and benzoquinone (0.5 equiv) in NMP, a wide range of olefins were coupled with various 2-nitrobenzoates at 130 °C with the release of carbon dioxide to afford the corresponding vinyl arenes in good yields.

## Introduction

The palladium-catalyzed coupling of olefins with aryl or vinyl substrates, developed by Mizoroki [1] and Heck [2–3] in the 1970s, is one of the most important, reliable and generally applicable reactions for carbon–carbon bond formation in organic synthesis. Over the last decade, several highly active catalyst systems have been developed for these reactions [4–5]. Usually, aryl halides are employed as starting materials [6–9], but other aryl sources such as aryl triflates [10–12], diazonium salts [13–14], arylsulfonyl halides [15] and aroyl chlorides [16] have also been used. Several protocols were recently described that draw on widely available carboxylic acid derivatives as alternative aryl sources. This not only extended the substrate basis of Heck reactions, but also opened up opportunities to reduce the problematic salt load of these transformations. In this context, De Vries developed decarbonylative Heck reactions of benzoic homoanhydrides – a salt-free process for Heck vinylations, which however, requires recycling of the arenecarboxylate leaving group [17–18]. We developed a process in which benzoic acids are activated by esterification with electron-poor phenols with the loss of water [19]. The esters are then subjected to a decarbonylative Heck reaction to yield vinyl arenes, CO and the phenols, which can be used in further reaction cycles. In a related approach, the benzoic acids activated as isopropenyl esters undergo a decarbonylative Heck reaction that yields the vinyl arenes along with CO and acetone [20]. Since no base is added, only volatile by-products are generated instead of waste salts, and the amount of solvent can be minimized. Myers and co-workers introduced another version of the Heck reaction in 2002, in which they converted mostly electron-rich aromatic carboxylic acids and alkenes to vinyl arenes with the release of CO_2_ [21–23]. This decarboxylative Heck reaction allows the direct conversion of benzoic acids without prior activation, but requires the addition of stoichiometric amounts of both base and an oxidant. In the original protocol, an excess of silver carbonate (3 equiv) was added to fulfil these functions. The work of Myers represents a milestone in the development of palladium-catalyzed decarboxylative coupling reactions. However, his original protocol suffers from several drawbacks, including the relatively narrow substrate scope, the high loadings of the Pd-catalyst, and the use of silver salts in over stoichiometric quantities. Very recently, Su published an improved procedure for this transformation, in which *p*-benzoquinone is employed as oxidant [24]. However, their protocol is strictly limited to highly activated benzoates. We herein disclose an alternative protocol for the Pd-catalyzed decarboxylative Heck reaction, in which copper(II) fluoride is utilized as the oxidant. This way, various nitrobenzoic acids are successfully coupled with several olefins to give the corresponding vinyl arenes in moderate to good yields.

## Results and Discussion

In our search for a silver-free catalyst system for decarboxylative Heck reactions, we chose the conversion of 2-nitrobenzoic acid (**1a**) with styrene (**2a**) to yield 2-nitro-stilbene (**3aa**) as a model reaction, and investigated the catalytic activity of various combinations of palladium precursors, additives and oxidants (Table 1). The choice of an electron-deficient benzoic acid was motivated by our intention to overcome substrate limitations observed in Myers’ original protocol, which can be understood on the basis of the proposed reaction mechanism. In his process, decarboxylation takes place at the palladium, and the role of the silver is restricted to acting as an oxidizing agent. Palladium has been found to be an effective mediator in the protodecarboxylation of particularly electron-rich, ortho-substituted benzoic acids, e.g. 2,6-dimethoxybenzoic acid, whereas electron-poor derivatives are not easily decarboxylated. The palladium-catalyzed protodecarboxylation reported by Kozlowski [25], and the palladium-catalyzed decarboxylative cross-couplings described by Forgione, Bilodeau and Liu [26–27] are limited to particularly activated, mostly 2,6-disubstituted or heterocyclic derivatives. We conclude from the fact that Myers’ protocol – in contrast to the silver-free version by Su – has a somewhat broader scope, that it allows for an additional catalytic cycle in which the decarboxylation occurs at the silver cocatalyst. Our desired decarboxylative Heck reaction of non-activated benzoates would have to proceed via this alternative catalytic cycle.

We started our search for an active catalyst using the system employed by Myers, and observed the formation of the desired product, albeit in the expected low yields. After some optimization, we were able to convert the electron-poor 2-nitrostilbene (**3aa**) in high yields even when lowering the palladium catalyst loading from 20 mol % to 5 mol %, and the amount of silver carbonate from 3 to 1.5 equivalents (entry 1). A further reduction to 1 or even 0.5 equivalents led to decreased yields (entries 2 and 3). We next investigated several palladium sources and found that palladium acetate gave yields comparable to the substantially more expensive palladium hexafluoroacetylacetonate (entry 4). In all reactions, nitrobenzene (**4a**) was formed as a byproduct. We assumed that it arises from a competing silver-catalyzed protodecarboxylation as also observed in decarboxylative cross-coupling reactions [28–33]. Control experiments confirmed that under these reaction conditions, 2-nitrobenzoic acid does not protodecarboxylate in the presence of palladium alone with or without an oxidant present, and that this side reaction is mediated by silver, irrespective of the presence of palladium (entries 5–8). In contrast, 2,6-dimethoxybenzoic acid can be decarboxylated both by 5 mol % palladium trifluoroacetate and by 5 mol % silver salts yielding 2,6-dimethoxybenzene in 75% and 99% yield, respectively. This finding further supports our theory that the catalyst cycle outlined by Myers for electron-rich benzoic acids, in which silver(I) acts as only an oxidant and is not involved in the decarboxylation step, may not hold true for electron-deficient benzoic acids. Instead, an additional decarboxylation catalyst is required to convert these substrates into the corresponding aryl metal species. Besides silver(I), we expected that copper salts should also mediate this step, as this metal has been used by Nilsson and Cohen in protodecarboxylation reactions [34–37]. Thus, we investigated several copper bases in place of silver carbonate. With copper(I) oxide and copper(II) carbonate, nitrobenzene (**4a**) was formed predominantly, while the desired Heck product was observed only in trace quantities (entries 9 and 10). When copper(II) fluoride was used, we obtained 27% of the product along with 48% of the undesired protodecarboxylation product (entry 11).

**Table 1:** Reaction of 2-nitrobenzoic acid with styrene.

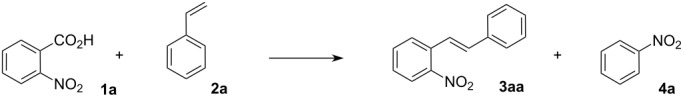						
**Entry**	**Pd.-cat. (mol %)**	**Oxidant (mmol)**	**Additive**	**Solvent**	**3aa [%]^a^**	**4a [%]^a^**

1	Pd(acac-F_6_)_2_ (5 mol %)	Ag_2_CO_3_ (1.5 mmol)	-	DMF/DMSO	52	29
2	"	Ag_2_CO_3_ (1.0 mmol)	-	"	45	31
3	"	Ag_2_CO_3_ (0.5 mmol)	-	"	14	24
4	Pd(OAc)_2_ (2 mol %)	Ag_2_CO_3_ (1.0 mmol)	-	"	57	22
5	-	Ag_2_CO_3_ (0.05 mmol)	-	"	0	56
6	Pd(OAc)_2_ (10 mol %)	-	-	"	0	0
7	Pd(CF_3_CO_2_)_2_ (5 mol %)	-	-	"	0	0
8	Pd(OAc)_2_ (2 mol %)	BQ (2.0 mmol)	-	"	0	0
9	"	Cu_2_O (1.0 mmol)	-	"	0	96
10	"	Cu_2_CO_3_ (1.0 mmol)	-	"	0	65
11	"	CuF_2_ (2.0 mmol)	-	"	27	48
12^b^	"	"	-	"	40	30
13^b^	"	CuF_2_ (0.5 mmol)	-	"	13^c^	65
14^b^	"	CuF_2_ (2.0 mmol)	3 Å MS^d^	"	44	16
15^b^	"	"	"	NMP	46	17
16^e^	"	"	" / phen.	"	60	18
17^e^	"	"	" / bipy.	"	59	17
18^e^	"	"	" / tan.	"	67	17
19^e^	"	" / NQ (0.5 mmol)	"	"	65	19
20^e^	"	" / BQ (0.5 mmol)	"	"	89	8

Conditions: 2-Nitrobenzoic acid (1.00 mmol), styrene (1.50 mmol), Pd-cat., oxidant, solvent (3 mL, 5% of DMSO), 20 h, 120 °C. ^a^Yields were determined by GC analysis, with *n*-tetradecane as internal standard; ^b^potassium 2-nitrobenzoate (1.00 mmol); ^c^along with 13% 2,2′-dinitrobiphenyl; ^d^3 Å MS (500 mg); ^e^potassium 2-nitrobenzoate (1.00 mmol), styrene (1.50 mmol), N-ligand (4 mol %). MS = molecular sieves, phen. = 1,10-phenanthroline, bipy. = 2,2′-bipyridine, tan. = 1,4,5-triazanaphthalene, NQ = 1,4-naphthoquinone, BQ = *p*-benzoquinone.

Even after careful exclusion of moisture and the use of preformed potassium 2-nitrobenzoate, the yields remained unsatisfactory (41%) and the protodecarboxylation could not effectively be suppressed (entry 12). Another unwanted byproduct, i.e. 2,2′-dinitrobiphenyl, appeared when reducing the quantity of the copper fluoride to less than 1 equivalent, which results from homocoupling of the arylcopper intermediate (entry 13).

The selectivity for the vinyl arene product was improved by adding molecular sieves to the reaction medium (entry 14). We were now able to switch from a DMF/DMSO mixture to the less toxic solvent *N*-methyl-2-pyrrolidone (NMP) without a decrease in yields (entry 15). Next, we investigated the influence of ligands on the performance of the catalyst system, and discovered that phosphines impede the decarboxylation step. In contrast, nitrogen-containing ligands had a beneficial effect (entries 16–18). In the presence of 1,4,5-triazanaphthalene, the Heck product was formed in 67% yield (entry 18). We subsequently tested several oxidants in an attempt to prevent the formation of palladium(0) species, which we assumed to be catalytically inactive in this process. This proved to be a good strategy, as the addition of *p*-benzoquinone resulted in higher yields of the Heck product, while at the same time suppressing the formation of the undesired nitrobenzene (**4a**) (entries 19 and 20). Thus, under optimized conditions, potassium 2-nitrobenzoate was decarboxylated and coupled with styrene in the presence of 2 mol % palladium acetate, 4 mol % 1,4,5-triazanaphthalene, 2 equivalents of copper fluoride, 0.5 equivalents of *p*-benzoquinone, and excess molecular sieves in NMP at 120 °C, yielding the vinyl arene **3aa** in 89%.

**Scheme 1:**
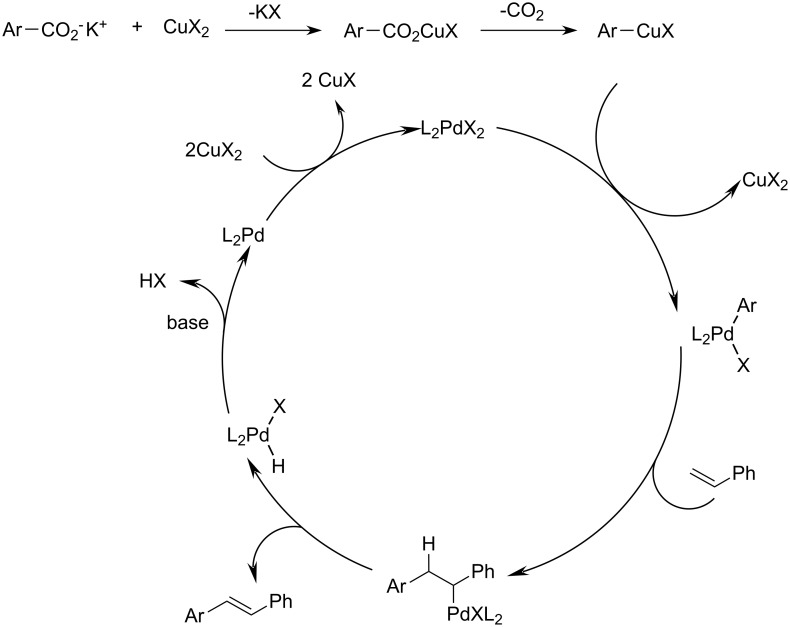
Proposed mechanism for the decarboxylative Heck reaction.

Based on our mechanistic studies of this and other decarboxylative couplings, we propose the mechanism detailed in Scheme 1. In the first step, the benzoate decarboxylates with formation of an aryl metal species. In contrast to Myers’ mechanism, which has been confirmed for electron-rich substrates [38], this step proceeds at a silver or copper mediator rather than at the palladium center. The resulting aryl residue is transferred to a Pd(II) center with formation of an arylpalladium species. Insertion of the olefin furnishes an alkylpalladium species which, after internal rotation, liberates the vinyl arene product via β-hydride elimination. The resulting palladium hydride species is converted back to the initial Pd(II) catalyst by reaction with the copper.

We next investigated the scope of the new protocol with regard to the olefin substrate. As shown in Table 2, styrenes, acrylate esters, and even acrylamides were coupled in good yields. In all cases, the reaction proceeded with high selectivities for the (*E*)-configured linear products. Other isomers were detected in trace amounts only.

**Table 2:** Variation of the olefin.

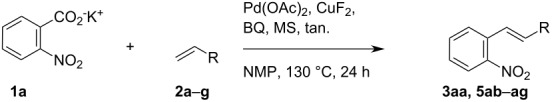			
**Entry**	**Olefin**	**Product**	**Yield [%]^a^**

1	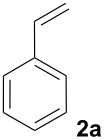	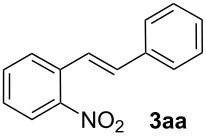	90
2	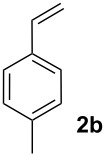	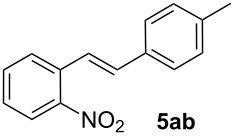	87
3	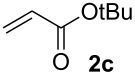	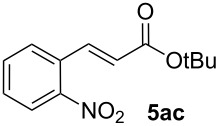	81
4	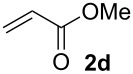	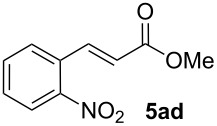	86
5	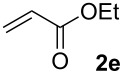	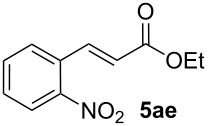	88
6	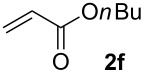	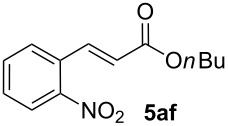	85
7	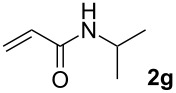	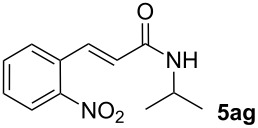	71

Conditions: Potassium 2-nitrobenzoate (1.50 mmol), olefin (1.00 mmol), copper(II) fluoride (2.00 mmol), palladium(II) acetate (0.02 mmol), 1,4,5-triazanaphthalene (0.04 mmol), *p*-benzoquinone (0.50 mmol), 3 Å molecular sieves (350 mg), NMP, 20 h, 130 °C; ^a^isolated yields.

Next, we examined the substrate range with regard to the carboxylate substrate and found that the new protocol is particularly suitable for 2-nitro-substituted carboxylates (Table 3). Various compounds of this type were converted to stilbenes in good yields. In contrast, arenecarboxylates with a nitro-group in meta- or para-position gave unsatisfactory results. Interestingly, substrates with 2-methoxy or 2-fluoro substituents, which are highly reactive in other decarboxylative couplings mediated by palladium alone, gave low yields in this process. Taking into account that 2-nitrobenzoates are among the best substrates for copper-mediated decarboxylation processes, this finding further supports our hypothesis that the decarboxylation takes place at the copper (entry 7 and 8).

**Table 3:** Variation of the benzoic carboxylate derivative.

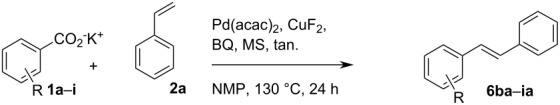			
**Entry**	**Carboxylate**	**Product**	**Yield [%]^a^**

1	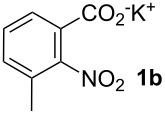	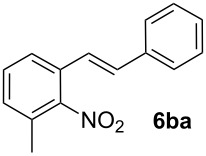	40
2	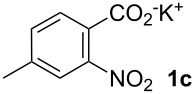	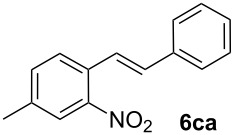	90
3	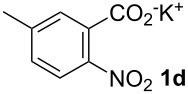	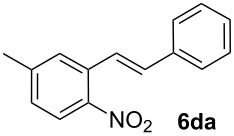	85
4	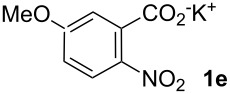	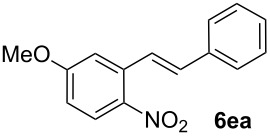	93
5	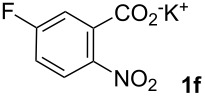	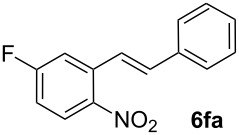	44
6	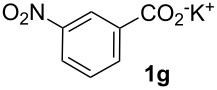	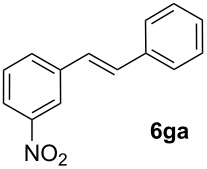	0
7	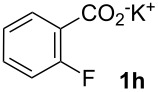	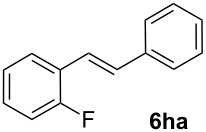	(7)
8	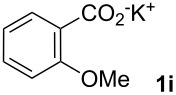	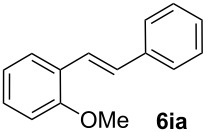	(2)

Conditions: Potassium carboxylate **1a**–**i** (1.50 mmol), styrene (1.00 mmol) copper(II) fluoride (2.00 mmol), palladium(II) acetate (0.02 mmol), 1,4,5-triazanaphthalene (4 mol %), *p*-benzoquinone (0.50 mmol), 3 Å molecular sieves (350 mg), NMP, 20 h, 130 °C. ^a^Isolated yields; yields in parentheses are GC-yields.

## Conclusion

In conclusion, we were able to show that decarboxylative Heck reactions can also be performed with carboxylic acids that cannot be decarboxylated at a palladium catalyst. The coupling of electron-poor 2-nitrobenzoates was achieved by combining a copper-mediated decarboxylation with a palladium-catalyzed Heck coupling. The new mechanistic pathway is likely to open up new perspectives for decarboxylative Heck reactions by overcoming some intrinsic substrate limitations. However, due to the fact that the decarboxylation of the benzoate and coupling of the resulting aryl residue now take place at a different metal center, the balance between the decarboxylation step and the remainder of the catalytic cycle becomes rather delicate. Current work is directed toward the development of new catalyst systems that would allow the coupling of a broader range of aromatic carboxylic acids.

## Experimental

### General remarks

Reactions were performed in oven-dried glassware under a nitrogen atmosphere containing a Teflon-coated stirrer bar and dry septum, unless otherwise specified. Solvents were purified by standard procedures prior to use. All reactions were monitored by GC using *n*-tetradecane as an internal standard. Response factors of the products with regard to *n*-tetradecane were obtained experimentally by analyzing known quantities of the substances. GC analyses were carried out using an HP-5 capillary column (Phenyl Methyl Siloxane 30 m × 320 × 0.25, 100/2.3-30-300/3) and a time program beginning with 2 min at 60 °C, followed by a 30 °C/min ramp to 300 °C, and then a 3 min hold at this temperature. Column chromatography was performed using a Combi Flash Companion-Chromatography-System (Isco-Systems) and RediSep packed columns (12 g).

### General procedure for the synthesis of the potassium carboxylates

A 250 mL, two-necked, round-bottomed flask was charged with the carboxylic acid (20 mmol) and ethanol (20 mL). To this, a solution of potassium *tert*-butoxide (2.24 g, 20 mmol) in ethanol (20 mL) was added dropwise over 2 h. After complete addition, the reaction mixture was stirred for a further 1 h at room temperature. The gradual formation of a white precipitate was observed. The resulting solid was collected by filtration through a 7-cm Büchner funnel, washed sequentially with ethanol (2 × 10 mL) and cold (0 °C) diethyl ether (10 mL), transferred to a round-bottomed flask, and dried at 2 × 10^−3^ mmHg to provide the corresponding potassium carboxylates **1a**–**i** in 70–98% yield.

### General procedure for the decarboxylative Heck vinylation

An oven dried 20 mL crimp-top vial equipped with a septum cap and a stirring bar was charged with potassium carboxylate **1a**–**i** (1.50 mmol), copper(II) fluoride (203 mg, 2.00 mmol), palladium(II) acetate (4.58 mg, 0.02 mmol), 1,4,5-triazanaphthalene (5.25 mg, 0.04 mmol), *p*-benzoquinone (54.0 mg, 0.50 mmol) and 3 Å molecular sieves (350 mg, powdered, and dried in the microwave). The reaction vessel was closed, evacuated and filled with nitrogen three times. A stock solution of the corresponding coupling partner **2a**–**g** (1.00 mmol) and the internal GC standard *n*-tetradecane (50 µL) in NMP (2.0 mL) was added with a syringe, and the resulting mixture stirred at 130 °C for 24 h. The reaction solution was then cooled to room temperature, diluted with ethyl acetate and filtered through Celite / SiO_2_. The solution was washed successively with aqueous HCl (1N, 20 mL), sodium hydrogen carbonate solution (20 mL), and brine (20 mL), dried over MgSO_4_, filtered, and the solvents removed in vacuo. Purification of the residue by column chromatography (SiO_2_, hexane / ethyl acetate gradient) gave the corresponding product.

**3-Methyl-2-nitrostilbene (6ba)** was synthesized from potassium 3-methyl-2-nitrobenzoate (**1b**) (329 mg, 1.50 mmol) and styrene (**2a**) (104 mg, 1.00 mmol). Purification by column chromatography (SiO_2_, hexane / ethyl acetate 4:1) gave **6ba** as a yellow oil (96 mg, 40%). ^1^H NMR (CDCl_3_, 400 MHz): δ = 7.59 (d, *J* = 8.1 Hz, 1H), 7.47 (d, *J* = 7.3 Hz, 2H), 7.33–7.39 (m, 3H), 7.30 (d, *J* = 7.1 Hz, 1H), 7.19 (d, *J* = 7.6 Hz, 1H), 7.10–7.17 (m, 1H), 6.92–6.99 (m, 1H), 2.33 (s, 3H) ppm; ^13^C NMR (101 MHz, CDCl_3_): δ = 136.4 (s), 130.1 (s), 130.0 (s), 129.7 (s), 128.8 (s), 128.6 (s), 127.0 (s), 124.1 (s), 121.1 (s), 17.3 (s) ppm; MS (EI), *m/z* (%): 222 (8), 207 (10), 193 (10), 152 (10), 133 (74), 104 (100); IR (NaCl): 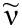 = 3027 (w), 1602 (w), 1524 (s), 1367 (s), 958 (m), 780 (m) cm^−1^; Anal. Calcd. for C_15_H_13_NO_2_: C = 75.30, H = 5.48, N = 5.85. Found C = 75.40, H = 5.43, N = 5.75.

**4-Methyl-2-nitrostilbene (6ca)** [CAS: 1054567-62-2] was synthesized from potassium 4-methyl-2-nitrobenzoate (**1c**) (329 mg, 1.50 mmol) and styrene (**2a**) (104 mg, 1.00 mmol). Purification by column chromatography (SiO_2_, hexane / ethyl acetate 4:1) gave **6ca** as a yellow oil (220 mg, 90%). ^1^H NMR (CDCl_3_, 400 MHz): δ = 7.91 (d, *J* = 8.5 Hz, 1H), 7.63 (d, *J* = 16.3 Hz, 1H), 7.51–7.56 (m, 3H), 7.35–7.40 (m, 2H), 7.31 (d, *J* = 7.2 Hz, 1H), 7.18 (d, *J* = 8.5 Hz, 1H), 7.05 (d, *J* = 16.0 Hz, 1H), 2.46 (s, 3H) ppm; ^13^C NMR (101 MHz, CDCl_3_): δ = 145.9 (s), 136.7 (s), 133.5 (s), 133.3 (s), 128.8 (s), 128.7 (s), 128.5 (s), 127.1 (s), 125.0 (s), 124.1 (s), 21.5 (s) ppm; MS (EI), *m/z* (%): 222 (32), 207 (19), 194 (24), 165 (20), 133 (100), 77 (45); IR (NaCl): 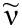 = 3059 (w), 2923 (w), 1605 (m), 1581 (m), 1515 (s), 1343 (s) cm^−1^.

**5-Methyl-2-nitrostilbene (6da)** [CAS: 861631-64-3] was synthesized from potassium 5-methyl-2-nitrobenzoate (**1d**) (329 mg, 1.50 mmol) and styrene (**2a**) (104 mg, 1.00 mmol). Purification by column chromatography (SiO_2_, hexane / ethyl acetate 4:1) gave **6da** as a yellow oil (205 mg, 85%). ^1^H NMR (CDCl_3_, 400 MHz): δ = 7.92 (d, *J* = 8.3 Hz, 1H), 7.66 (d, *J* = 16.1 Hz, 1H), 7.53–7.62 (m, 3H), 7.39–7.47 (m, 2H), 7.31–7.38 (m, 1H), 7.19 (d, *J* = 8.3 Hz, 1H), 7.08 (d, *J* = 16.1 Hz, 1H), 2.48 (s, 3H) ppm; ^13^C NMR (101 MHz, CDCl_3_): δ = 145.5 (s), 143.7 (s), 136.3 (s), 133.1 (s), 132.8 (s), 128.3 (s), 128.2 (s), 128.0 (s), 126.6 (s), 124.5 (s), 123.6 (s), 21.0 (s) ppm; MS (EI), *m/z* (%): 240 (12) [M^−^], 223 (16), 195 (31), 179 (15), 134 (89), 104 (100); IR (NaCl): 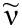 = 3060 (w), 3025 (w), 1605 (m), 1581 (m), 1514 (s), 1340 (s) cm^−1^.

**5-Methoxy-2-nitrostilbene (6ea)** [CAS: 879124-26-2] was synthesized from potassium 5-methoxy-2-nitrobenzoate (**1e**) (353 mg, 1.50 mmol) and styrene (**2a**) (104 mg, 1.00 mmol). Purification by column chromatography (SiO_2_, hexane / ethyl acetate 4:1) gave **6ea** as a yellow solid (238 mg, 93%). ^1^H NMR (CDCl_3_, 400 MHz): δ = 8.07 (d, *J* = 9.2 Hz, 1H), 7.72 (d, *J* = 16.0 Hz, 1H), 7.54 (d, *J* = 7.5 Hz, 2H), 7.35–7.43 (m, 2H), 7.32 (d, *J* = 7.5 Hz, 1H), 7.13 (d, *J* = 2.4 Hz, 1H), 7.01 (d, *J* = 16.0 Hz, 1H), 6.86 (dd, *J* = 8.9, 2.7 Hz, 1H), 3.92 (s, 3H) ppm; ^13^C NMR (101 MHz, CDCl_3_): δ = 163.2 (s), 141.1 (s), 136.5 (s), 136.3 (s), 133.7 (s), 128.8 (s), 128.6 (s), 127.7 (s), 127.2 (s), 126.0 (s), 124.9 (s), 114.0 (s), 113.2 (s), 113.0 (s), 56.0 (s) ppm; MS (EI), *m/z* (%): 238 (12), 165 (18), 149 (51), 121 (35), 106 (100), 77 (34); IR (KBr): 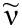 = 1579 (m), 1506 (s), 1333 (s), 1269 (m), 1232 (m); 1076 (m) cm^−1^; mp 65–66 °C.

**5-Fluoro-2-nitrostilbene (6fa)** was synthesized from potassium 5-fluoro-2-nitrobenzoate (**1f**) (335 mg, 1.50 mmol) and styrene (**2a**) (104 mg, 1.00 mmol). Purification by column chromatography (SiO_2_, hexane / ethyl acetate 9:1) gave **6fa** as a yellow solid (106 mg, 44%). ^1^H NMR (CDCl_3_, 400 MHz): δ = 8.04 (dd, *J* = 9.1, 5.0 Hz, 1H), 7.63 (d, *J* = 16.1 Hz, 1H), 7.54 (d, *J* = 7.3 Hz, 2H), 7.33–7.44 (m, 4H), 7.03–7.12 (m, 2H) ppm; ^13^C NMR (101 MHz, CDCl_3_): δ = 163.5 (s), 136.4 (s), 136.1 (s), 135.1 (s), 129.0 (s), 128.9 (s), 127.7 (s), 127.3 (s), 122.9 (s), 115.1 (s), 114.8 (s), 114.7 (s), 114.5 (s), 100.0 (s) ppm; MS (EI), *m/z* (%): 226 (32), 197 (17), 184 (13), 171 (16), 137 (100), 91 (32); IR (KBr): 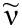 = 1616 (m), 1578 (m), 1597 (s), 1347 (m), 1272 (m), 952 (m) cm^−1^; Anal. Calcd. for C_14_H_10_FNO_2_: C = 69.13, H = 4.14, N = 5.76. Found C = 68.96, H = 4.30, N = 5.77; mp 66–67 °C.

See Supporting Information File 1 for details of the syntheses of the compounds **1a**–**i**, **3aa** and **5ab**–**ag**.

## Supporting Information

File 1 Experimental Section: The synthesis, purification and characterization data of all substances given in this article are provided in the Supporting Information.
