# Supplementary material for: Pd-catalyzed decarboxylative Heck vinylation of 2-nitrobenzoates in the presence of CuF2
Source: Beilstein J Org Chem. 2010 May 3;6:43. doi: 10.3762/bjoc.6.43 (PMC2887305; doi:10.3762/bjoc.6.43)
Supplement: File 1 — Experimental Section: The synthesis, purification and characterization data of all substances given in this article are provided in the Supporting Information. [file Beilstein_J_Org_Chem-06-43-s001.pdf]

# Supporting Information

# for

# Pd-catalyzed decarboxylative Heck vinylation of 2-nitro-benzoates in the presence of $CuF_2$

Lukas J. Gooßen*, Bettina Zimmermann, Thomas Knauber

Address: Department of Chemistry, Organic Chemistry, Technische Universität Kaiserslautern, Erwin-Schrödinger-Strasse, Geb. 54, D-67663 Kaiserslautern, Germany

Email: Lukas J. Gooßen* - goossen@chemie.uni-kl.de

* Corresponding author

## Experimental Section

### General remarks

Reactions were performed in oven-dried glassware under a nitrogen atmosphere containing a Teflon-coated stirrer bar and dry septum, unless otherwise specified. Solvents were purified by standard procedures prior to use. All reactions were monitored by GC using *n*-tetradecane as an internal standard. Response factors of the products with regard to *n*-tetradecane were obtained experimentally by analyzing known quantities of the substances. GC analyses were carried out using an HP-5 capillary column (Phenyl Methyl Siloxane 30 m × 320 × 0.25, 100/2.3-30-300/3) and a time program beginning with 2 min at 60 °C followed by 30 °C/min ramp to 300 °C

and then 3 min at this temperature. Column chromatography was performed using a Combi Flash Companion-Chromatography-System (Isco-Systems) and RediSep packed columns (12 g). NMR spectra were obtained on Bruker AMX 600 or 400 systems using $CDCl_3$, methanol-$d_4$ or $D_2O$ as solvent, with proton and carbon resonances at 600/400 and 151/100 MHz, respectively. Mass spectral data were acquired on a GC-MS Saturn 2100 T (Varian).

**General procedure for the synthesis of the potassium carboxylates**

A 250 mL, two-necked, round-bottomed flask was charged with the carboxylic acid (20.0 mmol) and ethanol (20 mL). To this, a solution of potassium *tert*-butoxide (2.24 g, 20 mmol) in ethanol (20 mL) was added dropwise over 2 h. After complete addition, the reaction mixture was stirred for a further 1 h at room temperature. The gradual formation of a white precipitate was observed. The resulting solid was collected by filtration through a 7-cm Büchner funnel, washed sequentially with ethanol (2× 10 mL) and cold (0 °C) diethyl ether (10 mL), transferred to a round-bottomed flask, and dried at $2 \times 10^{-3}$ mmHg to provide the corresponding potassium carboxylates **1a**–**i** in 70–98% yield.

*Potassium 2-nitrobenzoate (**1a**):* Starting from 2-nitrobenzoic acid (3.34 g, 20.0 mmol), compound **1a** [CAS: 15163-59-4] was obtained as a white powder (4.01 g, 98%). $^1$H NMR ($D_2O$, 600 MHz): $\delta$ = 8.02–8.06 (m, 1H), 7.71 (td, $J$ = 7.5, 1.2 Hz, 1H), 7.50–7.54 (m, 1H), 7.45 (dd, $J$ = 7.7, 1.3 Hz, 1H) ppm; $^{13}$C NMR ($D_2O$, 151 MHz): $\delta$ = 174.9 (s), 144.4 (s) 135.8 (s), 134.8 (s), 129.1 (s), 127.4 (s), 124.1 (s) ppm; Anal. Calcd. for $C_7H_4KNO_4$: C 40.9, H 1.9, N 6.8. Found C 40.9, H 1.7, N 6.9; mp 208–209 °C.

*Potassium 3-methyl-2-nitrobenzoate (**1b**):* Starting from 3-methyl-2-nitrobenzoic acid (3.62 g, 20.0 mmol), compound **1b** [CAS: 80841-44-7] was obtained as a white powder (4.09 g, 93%). $^{1}$H NMR (methanol-$d_4$, 600 MHz): $\delta$ = 7.57 (d, $J$ = 7.4 Hz, 1H), 7.30 (t, $J$ = 7.7 Hz, 1H), 7.24–7.27 (m, 1H), 2.19 (s, 3H) ppm; $^{13}$C NMR (methanol-$d_4$, 151 MHz): $\delta$ = 170.3 (s), 150.4 (s), 133.0 (s), 131.7 (s), 129.5 (s), 129.1 (s), 127.4 (s), 15.9 (s) ppm; Anal. Calcd. for $C_8H_6KNO_4$: C 43.8, H 2.7, N 6.4. Found C 43.8, H 2.7, N 6.3.

*Potassium 5-methyl-2-nitrobenzoate (**1d**):* Starting from 5-methyl-2-nitrobenzoic acid (3.62 g, 20.0 mmol), compound **1d** [CAS: 59639-92-8] was obtained as a white powder (4.27 g, 97%). $^{1}$H NMR (methanol-$d_4$, 600 MHz): $\delta$ = 784 (d, $J$ = 8.4 Hz, 1H), 7.36 (s, 1H), 7.28 (d, $J$ = 8.2 Hz, 1H), 2.42 (s, 3H) ppm; $^{13}$C NMR (methanol-$d_4$, 151 MHz): $\delta$ = 174.2 (s), 146.0 (s), 145.2 (s), 138.5 (s), 129.9 (s), 129.7 (s), 124.7 (s), 21.4 (s) ppm; Anal. Calcd. for $C_8H_6KNO_4$: C 43.8, H 2.7, N 6.4. Found C 43.7, H 2.8, N 6.4.

*Potassium 5-methoxy-2-nitrobenzoate (**1e**):* Starting from 5-methoxy-2-nitrobenzoic acid (1.38 g, 7.00 mmol) compound **1e** [CAS: 1071850-00-4] was obtained as a white powder (1.50 g, 91%). $^{1}$H NMR ($D_2O$, 600 MHz): $\delta$ = 8.17 (d, $J$ = 9.2 Hz, 1H), 7.07 (d, $J$ = 9.2 Hz, 1H), 7.00 (s, 1H), 3.96 (s, 3H) ppm; $^{13}$C NMR ($D_2O$, 151 MHz): $\delta$ = 174.7 (s), 164.4 (s), 139.0(s), 137.0 (s), 127.1 (s), 114.0 (s), 112.0 (s), 56.3 (s) ppm; Anal. Calcd. for $C_8H_6KNO_5$: C 40.85, H 2.57, N 5.95.Found C 40.69, H 2,87, N 5.79; mp 215–216 °C.

*Potassium 5-fluoro-2-nitrobenzoate (**1f**):* Starting from 5-fluoro-2-nitrobenzoic acid (561 mg, 3.00 mmol) compound **1f** [CAS: 92449-40-6] was obtained as a white powder (478 mg, 71%). $^{1}$H NMR ($D_2O$, 200 MHz): $\delta$ = 8.10–8.23 (m, 1H), 7.17–7.32

(m, 2H) ppm; $^{13}C$ NMR (methanol-$d_4$, 101 MHz): $\delta$ = 172.2 (s), 167.5 (s), 165.0 (s), 142.0 (s), 141.9 (s), 127.7 (s), 127.6 (s), 121.4 (s), 116.2 (s), 115.9 (s) ppm; Anal. Calcd. for $C_7H_3FKNO_4$: C 37.67, H 1.35, N 6.28. Found C 37.30, H 1.11, N 6.28; mp (decomposition): 229 °C.

*Potassium 3-nitrobenzoate (**1g**):* Starting from 3-nitrobenzoic acid (3.34 g, 20.0 mmol), compound **1g** [CAS: 18312-48-6] was obtained as a white powder (3.77 g, 92%). $^1H$ NMR ($D_2O$, 200 MHz): $\delta$ = 8.39 (s, 1H), 8.10 (dd, $J$ = 8.9, 2.1 Hz, 1H), 8.04 (d, $J$ = 7.8 Hz, 1H), 7.46 (t, $J$ = 8.0 Hz, 1H) ppm; $^{13}C$ NMR (methanol-$d_4$, 151 MHz): $\delta$ = 172.1 (s), 149.4 (s), 141.2 (s), 136.1 (s), 130.1 (s), 125.7 (s), 124.9 (s) ppm; Anal. Calcd. for $C_7H_4KNO_4$: C 40.9, H 1.9, N 6.8. Found C 40.7, H 1.9, N 6.7.

*Potassium 2-fluorobenzoate (**1h**):* Starting from 2-fluorobenzoic acid (2.80 g, 20.0 mmol), compound **1h** [CAS: 16463-37-9] was obtained as a white powder (3.39 g, 95%). $^1H$ NMR (methanol-$d_4$, 400 MHz): $\delta$ = 7.54 (td, $J$ = 7.5, 1.7 Hz, 1H), 7.20–7.27 (m, 1H), 7.02 (td, $J$ = 7.5, 1.0 Hz, 1H), 6.92–6.98 (m, 1H); $^{13}C$ NMR (methanol-$d_4$, 101 MHz): $\delta$ = 173.1 (s), 161.7 (d), 131.6 (d), 131.5 (s), 129.3 (d), 124.6 (d), 116.8 (d). Anal. Calcd. for $C_7H_4FKO_2$: C 47.2, H 2.2. Found C 47.0, H 2.2.

*Potassium 2-methoxybenzoate (**1i**):* Starting from 2-methoxybenzoic acid (3.04 g, 20.0 mmol) compound **1i** [CAS: 16463-34-6] was obtained as a white powder (2.65 g, 70%). $^1H$ NMR ($D_2O$, 400 MHz): $\delta$ = 7.28 (d, $J$ = 7.5 Hz, 2H), 6.97 (d, $J$ = 8.5 Hz, 1H), 6.90 (s, 1H), 3.72 (s, 3H) ppm; $^{13}C$ NMR ($D_2O$, 101 MHz): $\delta$ = 176.3 (s), 155.8 (s), 130.5 (s), 128.5 (s), 128.3 (s), 120.7 (s), 112.7 (s), 55.7 (s) ppm; mp 220 °C.

**General procedure for the decarboxylative Heck vinylation**

In an oven dried 20 mL crimp-top vial equipped with a septum cap and a stirring bar were placed the potassium carboxylate **1a**–**i** (1.50 mmol), copper(II)fluoride (203 mg, 2.00 mmol), palladium(II)acetate (4.58 mg, 0.02 mmol), 1,4,5-triazanaphthalene (5.25 mg, 0.04 mmol), *p*-benzoquinone (54.0 mg, 0.50 mmol) and 3 Å molecular sieves (350 mg, powdered and dried in the microwave). The reaction vessel was closed, evacuated and filled with nitrogen three times. A stock solution of the corresponding coupling partner **2a**–**g** (1.00 mmol) and the internal GC standard *n*-tetradecane (50 µL) in NMP (2.0 mL) was added with a syringe, and the resulting mixture stirred at 130 °C for 24 h. Then the reaction solution was cooled, diluted with ethyl acetate and filtered through Celite / $SiO_2$. The solution was washed successively with aqueous HCl (1N, 20 mL), saturated sodium hydrogen carbonate solution (20 mL) and then with brine (20 mL), dried over $MgSO_4$, filtered, and the solvents removed in vacuo. Purification of the residue by column chromatography ($SiO_2$, hexane / ethyl acetate gradient) gave the corresponding product.

*2-Nitrostilbene (**3aa**)* [CAS: 4264-29-3] was synthesised from potassium 2-nitrobenzoate (**1a**) (307 mg, 1.50 mmol), styrene (**2a**) (104 mg, 1.00 mmol) and copper(II)fluoride (152 mg, 1.50 mmol). Purification by column chromatography ($SiO_2$, hexane / ethyl acetate 4:1) gave **3aa** as an orange oil (220 mg, 90%). $^1$H NMR ($CDCl_3$, 600 MHz): $\delta$ = 7.95 (d, *J* = 8.2 Hz, 1H), 7.76 (d. *J* = 7.7 Hz, 1H). 7.57–7.61 (m, 2H), 7.54 (d, *J* = 7.7 Hz, 2H), 7.36–7.41 (m, 3H), 7.30–7.33 (m, 1H), 7.08 (d, *J* = 16.1 Hz, 1H) ppm; $^{13}$C NMR (151 MHz, $CDCl_3$): $\delta$ = 148.0 (s), 136.5 (s), 133.9 (s), 133.1 (2x, s), 129.4 (s), 128.9 (s), 128.7 (s), 128.2 (s), 128.0 (s), 127.1 (s), 124.8 (s), 123.6 (s) ppm; MS (EI), *m/z* (%): 208 (36), 180 (29), 165 (18), 152 (25), 92 (100), 77 (38); IR (NaCl): $\tilde{\nu}$ = 1522 (s), 1344 (m), 958 (m), 755 (m), 699 (w), 526 (w) $cm^{-1}$.

*2-Nitro-4'-Methylstilbene* ***(5ab)*** [CAS:823809-31-0] was synthesised from potassium 2-nitrobenzoate (**1a**) (307 mg, 1.50 mmol) and *p*-methyl styrene (**2b**) (118 mg, 1.00 mmol). Purification by column chromatography ($SiO_2$, hexane / ethyl acetate 4:1) gave **5ab** as an orange oil (209 mg, 87%). $^1H$ NMR ($CDCl_3$, 400 MHz): $\delta$ = 7.94 (d, *J* = 8.0 Hz, 1H), 7.75 (d. *J* = 7.8 Hz, 1H). 7.51–7.60 (m, 2H), 7.43 (d, *J* = 8.0 Hz, 2H), 7.34–7.40 (m, 1H), 7.19 (d, *J* = 7.8 Hz, 2H), 7.06 (d, *J* = 16.0 Hz, 1H), 2.37 (s, 3H) ppm; $^{13}C$ NMR (101 MHz, $CDCl_3$): $\delta$ = 148.1 (s), 138.7 (s), 133.9 (s), 133.8 (s), 133.2 (s), 132.9 (s), 129.5 (s), 128.0 (s), 127.7 (s), 127.1 (s), 124.7 (s), 122.4 (s), 21.3 (s) ppm; MS (EI), *m/z* (%): 222 (44), 207 (69), 194 (24), 119 (100), 92 (100), 77 (23); IR (NaCl): $\tilde{\nu}$ = 3023 (w), 1603 (m), 1519 (s), 1344 (s) 962 (m), 803 (m) $cm^{-1}$.

*2-Nitrocinnamic acid tert-butylester* ***(5ac)*** [CAS: 906552-00-9] was synthesised from potassium 2-nitrobenzoate (**1a**) (307 mg, 1.50 mmol) and *tert*-butyl acrylate (**2c**) (128 mg, 1.00 mmol). Purification by column chromatography ($SiO_2$, hexane / ethyl acetate 4:1) gave **5ac** as a beige solid (202 mg, 81%). $^1H$ NMR ($CDCl_3$, 400 MHz): $\delta$ = 7.98 (d, *J* = 15.8 Hz, 2H), 7.61 (d, *J* = 4.1 Hz, 2 H), 7.47–7.54 (m, 1H), 6.28 (d, *J* = 15.7 Hz, 1H), 1.52 (s, 9H) ppm; $^{13}C$ NMR (101 MHz, $CDCl_3$): $\delta$ = 165.0 (s), 148.5 (s), 138.6 (s), 133.3 (s), 130.8 (s), 130.0 (s), 129.1 (s), 125.4 (s), 124.8 (s), 81.1 (s), 28.2 (s) ppm; MS (EI), *m/z* (%): 176 (94), 147 (26), 130 (100), 104 (14), 76 (21), 57 (98); IR (KBr): $\tilde{\nu}$ = 2980 (w), 1703 (m), 1525 (m), 1342 (m), 1155 (m), 791 (w) $cm^{-1}$; mp 55–56 °C.

*2-Nitrocinnamic acid methyl ester* ***(5ad)*** [CAS: 39228-29-0] was synthesised from potassium 2-nitrobenzoate (**1a**) (307 mg, 1.50 mmol) and methyl acrylate (**2d**) (86.1 mg, 1.00 mmol). Purification by column chromatography ($SiO_2$, hexane / ethyl acetate 7:3) gave **5ad** as a brown solid (178 mg, 86%). $^1H$ NMR ($CDCl_3$, 400 MHz):

δ = 8.09 (d, *J* = 15.8 Hz, 1H), 8.02 (d, *J* = 7.8 Hz, 1H), 7.59–7.67 (m, 2H), 7.50–7.56 (m, 1H), 6.35 (d, *J* = 15.8 Hz, 1H), 3.81 (s, 3H) ppm; $^{13}$C NMR (101 MHz, $CDCl_3$): $\delta =$ 166.1 (s), 148.4 (s), 140.0 (s), 133.4 (s), 130.6 (s), 130.3 (s), 129.1 (s), 124.9 (s), 122.9 (s), 51.9 (s) ppm; MS (EI), *m/z* (%): 176 (32), 161 ( 53), 130 (88), 118 (34), 92 (100), 65 (66); IR (KBr): $\tilde{\nu}$ = 2953 (w), 1718 (s), 1636 (m), 1522 (s), 1346 (m), 1196 (m) cm$^{-1}$; mp 54–55 °C.

*2-Nitrocinnamic acid ethyl ester (**5ae**)* [CAS: 24393-59-7] was synthesised from potassium 2-nitrobenzoate (**1a**) (307 mg, 1.50 mmol) and ethyl acrylate (**2e**) (100 mg, 1.00 mmol). Purification by column chromatography ($SiO_2$, hexane / ethyl acetate 4:1) gave **5ae** as a light brown oil (196 mg, 88%). $^1$H NMR ($CDCl_3$, 400 MHz): δ = 7.99–8.10 (m, 2H), 7.60–7.65 (m, 2H), 7.49–7.55 (m, 1H), 6.34 (d, *J* = 15.7 Hz, 1H), 4.23–4.29 (m, 2H), 1.29–1.35 (m, 3H) ppm; $^{13}$C NMR (101 MHz, $CDCl_3$): $\delta =$ 165.7 (s), 148.4 (s), 133.4 (s), 130.6 (s), 130.2 (s), 129.1 (s), 124.8 (s), 123.4 (s), 60.9 (s), 14.2 (s) ppm; MS (EI), *m/z* (%): 176 (44), 147 (54), 130 (100), 92 (81), 76 (36), 65 (66); IR (NaCl): $\tilde{\nu}$ = 2983 (w), 1715 (s), 1639 (w), 1525 (s), 1346 (m), 1183 (m), 756 (m) cm$^{-1}$.

*2-Nitrocinnamic acid n-butyl ester (**5af**)* [CAS: 410074-36-1] was synthesised from potassium 2-nitrobenzoate (**1a**) (307 mg, 1.50 mmol) and *n*-butyl acrylate (**2f**) (128 mg, 1.00 mmol). Purification by column chromatography ($SiO_2$, hexane / ethyl acetate 7:3) gave **5af** as a brown oil (211 mg, 85%). $^1$H NMR ($CDCl_3$, 400 MHz): δ = 7.70–7.77 (m, 1H), 7.31–7.36 (m, 2H), 7.20–7.26 (m, 1H), 6.05 (d, *J* = 15.8 Hz, 1H), 3.91 (t, *J* = 6.7 Hz, 2H), 1.33–1.42 (m, 2H), 1.07–1.17 (m, 2H), 0.62–0.67 (m, 3H) ppm; $^{13}$C NMR (101 MHz, $CDCl_3$): $\delta =$ 165.5 (s), 148.1 (s), 140.6 (s), 139.4 (s), 133.1 (s), 133.0 (s), 132.7 (s), 130.8 (s), 130.4 (s), 129.9 (s), 128.8 (s), 128.6 (s), 124.6 (s),

124.9 (s), 123.3 (s), 122.1 (s), 121.2 (s)l 64.5 (s), 30.4 (s), 18.9 (s), 13.4 (s) ppm; MS (EI), *m/z* (%): 207 (10), 176 (34), 147 (61), 130 (100), 119 (23), 76 (25); IR (NaCl): $\tilde{\nu}$ = 2960 (m), 2873 (w), 1716 (s), 1526 (s), 1346 (s), 1180 (s) $cm^{-1}$.

*N-Isopropyl-2-nitrocinnamide* ***(5ag)*** [CAS: 159534-85-7] was synthesised from potassium 2-nitrobenzoate (**1a**) (307 mg, 1.50 mmol) and *N*-isopropyl acrylamide (**2g**) (113 mg, 1.00 mmol). Purification by column chromatography ($SiO_2$, hexane / ethyl acetate 1:4) gave **5ag** as a light brown solid (167 mg, 71%). $^1H$ NMR ($CDCl_3$, 400 MHz): $\delta$ = 7.93–8.02 (m, 2H), 7.57–7.64 (m, 2H), 7.47–7.54 (m, 1H), 6.35 (d, *J* = 15.5 Hz, 1H), 4.22 (d, *J* = 7.8 Hz, 1H), 1.23 (d, J = 6.5 Hz, 6H) ppm; $^{13}C$ NMR (101 MHz, $CDCl_3$): $\delta$ = 164.0 (s), 148.4 (s), 135.5 (s), 133.2 (s), 131.2 (s); 129.6 (s), 129.1 (s), 126.7 (s), 124.7 (s), 120.0 (s), 41.8 (s), 22.7 (s) ppm; MS (EI), *m/z* (%): 235 (4), 188 (44), 176 (47), 130 (100), 102 (33), 77 (20); IR (KBr): $\tilde{\nu}$ = 3300 (w), 2965 (w), 1654 (m), 1623 (m), 1522 (m), 1339 (m) $cm^{-1}$; mp 125–126 °C.

*3-Methyl-2-nitrostilbene* ***(6ba)*** was synthesised from potassium 3-methyl-2-nitrobenzoate (**1b**) (329 mg, 1.50 mmol) and styrene (**2a**) (104 mg, 1.00 mmol). Purification by column chromatography ($SiO_2$, hexane / ethyl acetate 4:1) gave **6ba** as a yellow oil (96 mg, 40%). $^1H$ NMR ($CDCl_3$, 400 MHz): $\delta$ = 7.59 (d, *J* = 8.1 Hz, 1H), 7.47 (d, *J* = 7.3 Hz, 2H), 7.33–7.39 (m, 3H), 7.30 (d, *J* = 7.1 Hz, 1H), 7.19 (d, *J* = 7.6 Hz, 1H), 7.10–7.17 (m, 1H), 6.92–6.99 (m, 1H), 2.33 (s, 3H) ppm; $^{13}C$ NMR (101 MHz, $CDCl_3$): $\delta$ = 136.4 (s), 130.1 (s), 130.0 (s), 129.7 (s), 128.8 (s), 128.6 (s), 127.0 (s), 124.1 (s), 121.1 (s), 17.3 (s) ppm; MS (EI), *m/z* (%): 222 (8), 207 (10), 193 (10), 152 (10), 133 (74), 104 (100); IR (NaCl): $\tilde{\nu}$ = 3027 (w), 1602 (w), 1524 (s), 1367 (s), 958 (m), 780 (m) $cm^{-1}$; Anal. Calcd. for $C_{15}H_{13}NO_2$: C = 75.30, H = 5.48, N = 5.85. Found C = 75.40, H = 5.43, N = 5.75.

*4-Methyl-2-nitrostilbene (**6ca**)* [CAS: 1054567-62-2] was synthesised from potassium 4-methyl-2-nitrobenzoate (**1c**) (329 mg, 1.50 mmol) and styrene (**2a**) (104 mg, 1.00 mmol). Purification by column chromatography ($SiO_2$, hexane / ethyl acetate 4:1) gave **6ca** as a yellow oil (220 mg, 90%). $^1$H NMR ($CDCl_3$, 400 MHz): $\delta$ = 7.91 (d, $J$ = 8.5 Hz, 1H), 7.63 (d, $J$ = 16.3 Hz, 1H), 7.51–7.56 (m, 3H), 7.35–7.40 (m, 2H), 7.31 (d, $J$ = 7.2 Hz, 1H), 7.18 (d, $J$ = 8.5 Hz, 1H), 7.05 (d, $J$ = 16.0 Hz, 1H), 2.46 (s, 3H) ppm; $^{13}$C NMR (101 MHz, $CDCl_3$): $\delta$ = 145.9 (s), 136.7 (s), 133.5 (s), 133.3 (s), 128.8 (s), 128.7 (s), 128.5 (s), 127.1 (s), 125.0 (s), 124.1 (s), 21.5 (s) ppm; MS (EI), *m/z* (%): 222 (32), 207 (19), 194 (24), 165 (20), 133 (100), 77 (45); IR (NaCl): $\tilde{\nu}$ = 3059 (w), 2923 (w), 1605 (m), 1581 (m), 1515 (s), 1343 (s) $cm^{-1}$.

*5-Methyl-2-nitrostilbene (**6da**)* [CAS: 861631-64-3] was synthesised from potassium 5-methyl-2-nitrobenzoate (**1d**) (329 mg, 1.50 mmol) and styrene (**2a**) (104 mg, 1.00 mmol). Purification by column chromatography ($SiO_2$, hexane / ethyl acetate 4:1) gave **6da** as a yellow oil (205 mg, 85%). $^1$H NMR ($CDCl_3$, 400 MHz): $\delta$ = 7.92 (d, $J$ = 8.3 Hz, 1H), 7.66 (d, $J$ = 16.1 Hz, 1H), 7.53–7.62 (m, 3H), 7.39–7.47 (m, 2H), 7.31–7.38 (m, 1H), 7.19 (d, J = 8.3 Hz, 1H), 7.08 (d, J = 16.1 Hz, 1H), 2.48 (s, 3H) ppm; $^{13}$C NMR (101 MHz, $CDCl_3$): $\delta$ = 145.5 (s), 143.7 (s), 136.3 (s), 133.1 (s), 132.8 (s), 128.3 (s), 128.2 (s), 128.0 (s), 126.6 (s), 124.5 (s), 123.6 (s), 21.0 (s) ppm; MS (EI), *m/z* (%): 240 (12) [$M^-$], 223 (16), 195 (31), 179 (15), 134 (89), 104 (100); IR (NaCl): $\tilde{\nu}$ = 3060 (w), 3025 (w), 1605 (m), 1581 (m), 1514 (s), 1340 (s) $cm^{-1}$.

*5-Methoxy-2-nitrostilbene (**6ea**)* [CAS:879124-26-2] was synthesised from potassium 5-methoxy-2-nitrobenzoate (**1e**) (353 mg, 1.50 mmol) and styrene (**2a**) (104 mg, 1.00 mmol). Purification by column chromatography ($SiO_2$, hexane / ethyl acetate 4:1) gave **6ea** as a yellow solid (238 mg, 93%). $^1$H NMR ($CDCl_3$, 400 MHz): $\delta$ = 8.07

(d, $J$ = 9.2 Hz, 1H), 7.72 (d, $J$ = 16.0 Hz, 1H), 7.54 (d, $J$ = 7.5 Hz, 2H), 7.35–7.43 (m, 2H), 7.32 (d, $J$ = 7.5 Hz, 1H), 7.13 (d, $J$ = 2.4 Hz, 1H), 7.01 (d, $J$ = 16.0 Hz, 1H), 6.86 (dd, $J$ = 8.9, 2.7 Hz, 1H), 3.92 (s, 3H) ppm; $^{13}$C NMR (101 MHz, $CDCl_3$): $\delta$ = 163.2 (s), 141.1 (s), 136.5 (s), 136.3 (s), 133.7 (s), 128.8 (s), 128.6 (s), 127.7 (s), 127.2 (s), 126.0 (s), 124.9 (s), 114.0 (s), 113.2 (s), 113.0 (s), 56.0 (s) ppm; MS (EI), *m/z* (%): 238 (12), 165 (18), 149 (51), 121 (35), 106 (100), 77 (34); IR (KBr): $\tilde{\nu}$ = 1579 (m), 1506 (s), 1333 (s), 1269 (m), 1232 (m); 1076 (m) cm$^{-1}$; mp 65–66 °C.

*5-Fluoro-2-nitrostilbene **(6fa)*** was synthesised from potassium 5-fluor-2-nitrobenzoat (**1f**) (335 mg, 1.50 mmol) and styrene (**2a**) (104 mg, 1.00 mmol). Purification by column chromatography ($SiO_2$, hexane / ethyl acetate 9:1) gave **6fa** as a yellow solid (106 mg, 44%). $^{1}$H NMR ($CDCl_3$, 400 MHz): $\delta$ = 8.04 (dd, $J$ = 9.1, 5.0 Hz, 1H), 7.63 (d, $J$ = 16.1 Hz, 1H), 7.54 (d, $J$ = 7.3 Hz, 2H), 7.33–7.44 (m, 4H), 7.03–7.12 (m, 2H) ppm; $^{13}$C NMR (101 MHz, $CDCl_3$): $\delta$ = 163.5 (s), 136.4 (s), 136.1 (s), 135.1 (s), 129.0 (s), 128.9 (s), 127.7 (s), 127.3 (s), 122.9 (s), 115.1 (s), 114.8 (s), 114.7 (s), 114.5 (s), 100.0 (s) ppm; MS (EI), *m/z* (%): 226 (32), 197 (17), 184 (13), 171 (16), 137 (100), 91 (32); IR (KBr): $\tilde{\nu}$ = 1616 (m), 1578 (m), 1597 (s), 1347 (m), 1272 (m), 952 (m) cm$^{-1}$; Anal. Calcd. for $C_{14}H_{10}FNO_2$: C = 69.13, H = 4.14, N = 5.76. Found C = 68.96, H = 4.30, N = 5.77; mp 66–67 °C.
